# Linking a research register to clinical records in older adults’ mental health services: a mixed-methods study

**DOI:** 10.1186/s13195-015-0103-8

**Published:** 2015-04-01

**Authors:** Dan Robotham, Joanne Evans, Andrew Watson, Iain Perdue, Thomas Craig, Diana Rose, Til Wykes

**Affiliations:** King’s College London, Institute of Psychiatry, Henry Wellcome Building, De Crespigny Park, London, SE5 8AF UK; UCL, Institute of Neurology, 33 Queen Square, London, WC1N 3BG UK

## Abstract

**Introduction:**

Patients can provide consent to have their clinical records linked to a research register, a process known as consent for contact (C4C). There is evidence about how to engage people with mental illness in C4C, but nothing specific to older adults. This is a priority area for research (for example, dementia trials), although sign-up rates to C4C are lower than for younger populations. Through this study we seek to understand these disparities.

**Methods:**

This was a two-stage cross-sectional observational study. In phase one, focus groups with service users, carers and clinicians informed a framework for clinicians to explain C4C to those on their caseload. In phase two, clinicians explained C4C to 26 service users (and carers where applicable). These conversations were recorded, and their content was analysed. Service users and carers were then interviewed to provide further feedback on their conversations with clinicians. A total of 31 service users, 24 carers and 13 clinical staff took part across the two phases.

**Results:**

In phase one, service users and carers sought assurance of the right to refuse participation in further studies (after joining C4C). Clinicians expressed concerns over legal and practical implications of ascertaining mental capacity and best interest. In phase two, clinicians’ explanations were less thorough than similar explanations given to younger adults with psychosis. Clinicians omitted details of service users’ right to stipulate contact arrangements, which was significantly associated with whether service users/carers agreed to join. Common reasons for joining C4C included altruism and the chance to speak to new people. Few participants refused to join, but reasons included avoidance of stress (potentially alleviated through the presence of a carer).

**Conclusions:**

Implementing C4C in older adults’ services requires clinicians to deliver concise, simple explanations to individuals and their carers where applicable. Older adults can be suspicious of unsolicited contact; thus, explanations must emphasise freedom to negotiate suitable contact arrangements. Hearing about research opportunities can be in the best interests of older adults, but communicating these opportunities requires a tailored approach.

## Introduction

Patient Electronic Health Records can improve the speed of research. Researchers can screen and identify potential research participants based upon clinical and diagnostic information. These potential participants can then be contacted and invited to take part in studies. This procedure accords with the UK National Health Service (NHS) Constitution, which promises to inform patients of relevant research opportunities [1].

Linking a patient record to a research register cannot happen without gaining the patient’s informed consent, which is dependent on them receiving an explanation of how the register and their record may be used. Clinicians are well placed to offer such explanations since they have an existing relationship with the patient and knowledge of the content of the record.

The South London and Maudsley NHS Trust developed a Consent for Contact (C4C) register through which service users can be informed about upcoming research [2,3]. Information is stored securely by the local healthcare organisation as an anonymous copy of patients’ clinical records. Researchers who wish to access the information must apply to de-anonymise the records of those patients who have opted-in to the register. Access to C4C is monitored by an oversight committee that includes a mixture of clinicians, service users, and research and information governance experts. This model has been described as ‘an approach that allows appropriate individuals to be identified and approached to take part, without giving researchers direct access to identifiable information before consent is obtained’ [4]. C4C follows an opt-in model; that is, patients must agree before joining the register. This differentiates it from controversial healthcare data sharing schemes that have used opt-out models; for example, Care.data in the UK [5].

Clinicians working in services for people with psychosis were trained in how to explain C4C to service users using a checklist. Three items on the checklist related to whether service users agreed to join the register: that service users would not be obliged to participate in future studies; that service users could come off the register at any time; and that service users could stipulate how, when, and how often they wished to be contacted from the register.

One area in which recruitment to C4C has proven difficult is in older adults’ mental health services. At the time of writing (16 October 2014) only 52% (*n* = 482) of older adults who had been asked to join the C4C register had agreed to join, compared with 68% (*n* = 1,048) in services for younger adults with psychosis and 71% (*n* = 4,947) across the total sample of mental health service users.

Recruitment to older adults’ mental health research is important. The societal and financial cost of dementia is estimated to be greater than the combined cost of heart disease, cancer, and stroke [6]. Anecdotal evidence suggests that research registers have improved recruitment to dementia research [7,8]. However, these registers are not linked to Electronic Health Records and therefore do not allow screening for the clinical characteristics of potential participants. This study assimilates existing knowledge of how clinicians explain C4C, and seeks to understand the challenges of recruiting older adults and how C4C could be better explained to this population.

## Methods

### Design

This is a two-stage cross-sectional observational study. The first phase involved developing training materials for clinicians with adaptations specifically to explain C4C to older adults and their carers. The second phase involved a recorded explanatory interview about C4C between trained clinicians and service users (with carers included as appropriate), followed by an independent interview between the researcher and the service user/carer. The content of the explanatory interview was analysed, and linked to whether the service user decided to join the register, using both quantitative and qualitative methods.

### Setting and participants

In phase one, three focus groups were held, one each with services users (*n* = 5), carers (*n* = 5), and clinical staff (*n* = 7). Service users and carers were recruited from memory clinics and community services in South London. Service users were included if they had a diagnosis of a dementia-related illness; carers were included if they were a primary carer for someone with a dementia-related illness. Staff participants were recruited from two inpatient wards and one community service for people with dementia. Consent was provided by all who participated in the focus groups.

In phase two, 26 service users completed the study. The service users were included if they were being treated by older adults’ mental health services in memory clinics, outpatient services, and inpatient wards. Their age ranged from 65 to 92 (mean = 78 years), 15 were women and 11 were men. Of the service user participants, 18 had a carer, one participant had two carers, and seven had no carer. In each case where the service user had a carer, the carer was also recruited. The participants had C4C explained to them by one of six trained clinicians. The majority of conversations took place in outpatient settings (*n* = 22), two in hospital and two in the home. Consent to participate was provided by all participants involved in the explanatory interview (that is, service users, carers and clinicians). The study was approved by the National Research Ethics Service, London Dulwich (reference: 11/LO/1255).

### Materials

Clinical, professional, and demographic information was collected. For service users this included diagnosis and time spent in contact with mental health services and with their care team. For carers, this included relationship with the service user and years spent as a carer. For staff participants, this included their role and years spent in the role.

In phase one, topic guides were created for respective focus groups with staff, service users, and carers. These guides were based on those created for people using psychosis services. Topic guides described the process of creating an Electronic Health Record-linked research register, its implications for research, carers’ involvement, mental capacity, decision-making, and when to invite people to join the register.

In phase two, clinicians received a 16-item explanation framework for C4C (Table 1), adapted from the earlier study. The primary outcome measure was agreement to join the C4C register. Variables analysed against the primary outcome included: the mention of key items in clinicians’ explanations; whether the decision to join C4C was made by the service user, the carer, or both; how explanations compared with explanations given by clinicians in psychosis services for younger adults; and why people agreed or refused to join the register.

**Table 1:** **Content of consultations between clinicians and service users/carers**

**Item**	**Clinician mentioned**	**Service user mentioned in consultation to researcher**	**Carer mentioned in consultation to researcher**
	**(total potential** ***n*** **= 26)**	**(total potential** ***n*** **= 26)**	**(total potential** ***n*** **= 20)**
Having an (electronic) health record	11	0	0
Benefits of research	22	0	0
Types of research	4	0	0
Personalised example of research	13	0	3
Researchers have been ‘approved’ (by regulatory bodies)	2	0	0
Researchers’ confidentiality	17	1	0
Researchers will identify you from the EHR	12	0	4
Researchers may contact you in future	22	4	7
C4C is voluntary	12	0	4
**Future studies are voluntary** ^**a**^	**15**	0	5
**Service user/carer can un-join the register (change their mind)** ^**a**^	**10**	3	3
Decision will not affect care	4	1	2
**Can agree contact arrangements; that is, what/when/how contacted** ^**b**^	**8**	2	3
Ask whether they wish to join the register	22	6	4
Questions and concerns	14	1	0
Who to contact for further information	5	2	5

C4C, Consent for Contact; EHR, Electronic Health Record. Bold data are significant. ^a^Marked a significant difference from whether younger adults joined a similar register. ^b^Marked a significant difference from whether younger adults and older adults joined the register.

### Procedure

In phase one, focus groups were conducted by a facilitator, assisted by a co-facilitator. They were audio-recorded and later transcribed. Staff focus groups were conducted at their place of work. Service user and carer focus groups were conducted either in ward settings or appropriate community venues.

In phase two, researchers enlisted clinicians working in older adults’ mental health services. Clinicians were trained using the C4C explanation framework, and were given an *aide memoire* of how to explain C4C to service users (adapted following phase one). Clinicians then asked service users/carers on their caseload whether they would like to join C4C, and gave the service user/carer an information leaflet to keep. Consent was obtained from service users and carer(s) in order to record explanations received from the clinician.

Immediately after the consultation, a researcher interviewed the service user and carer without the clinician present. The researcher asked whether they had understood the explanation in response to a set of standardised questions (these feedback interviews were also audio-recorded and transcribed).

### Data analysis

Phase one data from the focus groups were coded in NVivo 10 (QSR International, Melbourne, Victoria, Australia), and then thematically analysed separately for each of the participant groups. The topic guide was used as the basis for a coding frame. Two researchers used the frame to independently analyse the data. The frame was refined after coding all themes and subthemes into NVivo. The data from all three focus groups were then analysed as a whole.

For phase two, the content of each consultation between clinician and service user/carer was coded into NVivo 10 against the explanation framework. Two independent researchers coded the data. For consultations involving carers, the two coders ascertained and agreed whether the decision to join the register was made by the service user, was made by the carer, or was a joint decision. This was based on two factors: whether the clinician addressed the service user or carer; and the party who answered the question relating to whether they wanted to join the register (service user or carer).

Previous research suggested that three items on the checklist were of particular importance: the fact that future studies offered through C4C are voluntary; that the service user can change their mind about being on C4C; and that the service user could agree about what/when/how they wished to be contacted. To test the hypothesis that these items were predictive of agreement to join the register, one-sided chi-squared tests (in Statistical Package for the Social Sciences; IBM Corporation, Armonk, New York, US) were used to analyse whether sign-up rates to C4C differed according to clinicians’ explanations.

For secondary analyses, two-sided chi-squared tests were used to analyse: whether the remainder of the items on the explanation framework related to agreement rates; whether the decision to join C4C was made by the service user, the carer, or both; and whether clinicians in older adults’ services differed in their explanations to clinicians in psychosis services.

The data from the feedback interviews between service user/carer and the researcher were then analysed thematically to understand common motivations for joining C4C or not. Themes from the consultations were coded into NVivo and analysed by two independent researchers using the same process as in phase one.

## Results

### Participants

Service user and carer focus groups each included four women and one man. The clinician focus group included six women and one man; six worked on inpatient wards and one in community services, and two members were senior nurses and five were psychiatrists.

Sample demographics for phase two are presented in Table 2. Service users (*n* = 26) were predominantly women (58%) with a diagnosis related to Alzheimer’s disease or dementia (73%). Nineteen service users (73%) had carers, but there were 20 carers in total (one service user had two carers).

**Table 2:** **Service user and carer demographic data for phase two**

**Service users**			**Carers**			**Staff**	
	***n***	**%**		***n***	**%**		***n***
Male	11	42	Male	8	40	Male	2
Female	15	58	Female	12	60	Female	4
Ethnicity			Ethnicity			Ethnicity	
White British	12	46	White British	12	60	White British	2
Other	14	54	Other	8	40	Other	4
Diagnosis			Relationship			Role	
Dementia	19	73	Husband/wife	8	20	Doctor	3
Depression	3	12	Son/daughter	9	45	Nurse	1
Anxiety	2	8	Other	3	15	Care coordinator	2
Bipolar	1	4	Time as carer			Years in role	
Schizophrenia	1	4	< 1 year	13	65	< 1 year	0
Has a carer			1 to 5	2	10	1 to 9	4
Yes	19	73	6+	1	5	10+	1
No	7	27	Unknown	4	20	Unknown	1
Time with care team							
< 1 year	16	61					
> 1	10	38					
Time in mental health services							
< 1 year	1	3					
1 to 5	18	69					
6+	7	27					
Total	26			20			6

#### Service user and carer focus groups

Service users and carers wanted to know whether they could refuse participation in future studies once joining C4C. This should be seen in terms of competing commitments and limitations on time and energy, particularly for carers. Service users and carers distinguished between joining C4C and signing up to participate in future studies. One service user mentioned receiving nuisance telephone calls, and thus was reluctant to provide their telephone number.

“You then have an opportunity to say, sorry that is not my thing, is that right? (Service user focus group, #2)”

“We’re never pushed anyway, are we?” (Service user focus group, #5)

“My wife, was very keen at one stage for the brain scan but by the time the researcher came and asked us about it, she was not keen on it. […] I don’t see any reason why there shouldn’t be an approach, provided that there is the option to say no.” (Carer focus group, #2)

“I don’t think you should be bombarded, last year we had several and you just said no, no we were doing something at the moment and that was it but I don’t like saying no but in the end your whole life is revolving round […] Appointments … So you’ve got to look at it from the carers’ point of view that they’ve got a life, so to speak.” (Carer focus group, #3)

“I don’t want everybody knows my telephone number … I even have to take it off the book because they kept phoning me.” (Service user focus group, #4)

Carers’ reservations about joining C4C depended upon an acknowledgement of their commitments. Some may be in full-time employment; others may be older and/or unable to take on extra responsibilities due to mobility problems (also mentioned by one service user). For this reason, it is often necessary to provide assurance that future research studies will account for mobility requirements:

“What about the older carers though? Is there not a problem of the blind leading the blind? That the carer might not actually be very much more capable than the person they’re caring for and I think that it a potential problem.” (Carer focus group, #5)

“My wife is physically weak but mentally still very strong whilst I’m vice versa. […] I am physically OK but mentally weak.” (Service user focus group, #3)

#### Clinician focus groups

A main theme from the clinicians’ focus group was the legal implications of asking service users to join C4C. Perceived problems included capacity to consent, the challenge of identifying third parties who could provide permission, and ascertaining whether joining C4C would be in service users’ best interest.

“I think it would be important if they have their carers involved that they’re made aware of this approach, because we get complaints about things like that.” (Staff focus group, #3, inpatient)

“I can’t think of any patients at the moment that would be able to give capacity for consent so it would be a very involved process going through that, finding out who … would be the best person to ask, I can see it becoming fairly complicated. […] I wouldn’t be as comfortable making a best interest decision about going onto a register like this as it’s not as clearly a best interest decision in my mind, it’s in the Trust’s best interest.” (Staff focus group, #7, community)

#### Consultations

Table 1 shows how clinicians explained C4C to service users and carer(s) according to the explanation framework. The majority of these consultations lasted between 3 and 6 minutes (*n* = 15), but ranged from as brief as 1 minute to as long as 10 minutes. Table 1 also reports the number of times service users and carers recalled each aspect of the explanation (when feeding back to the researcher).

#### Agreement to join Consent for Contact

Nineteen out of the 26 consultations resulted in service users/carers agreeing to join C4C (73%). Only one factor in the explanation framework was related to agreement rates: whether service users/carers could stipulate how, when, and how often to be contacted. The difference was significant (χ = 4.3, degrees of freedom = 1, *P* = 0.048).

The service user was the primary decision maker in one-half of the consultations (*n* = 13) and the carer in eight consultations. The remaining five consultations displayed shared decision-making between service user and carer. When the carer was the primary decision-maker, agreement to join C4C was more likely than when service users were involved in the decision (either as primary or joint decision-maker; *n* = 18). This finding did not reach conventional statistical significance (χ = 4.3, degrees of freedom = 1. *P* = 0.06).

#### Comparisons with other clinical areas

Clinicians’ explanations differed in comparison with those given to younger adults. Clinicians within older adults’ services offered simpler explanations. They were less likely to explain that the service user could change their mind and leave C4C, or could stipulate how they would like to be contacted (the latter of which may affect agreement rates). Other lesser mentioned items included the fact that C4C was voluntary, that C4C offered people the chance to participate in different types of research, that any researchers using C4C would have been regulated before use, and where the service user could go for further information. Table 3 shows how the explanations differed.

**Table 3:** **Differences between explanations given to older adults and those given to younger adults**

**Item**	**Older adults (%)**	**Younger adults (using psychosis services) (%)**	**Significance**
Having an (electronic) health record	42	64	χ = 4, df = 1, *P* = 0.07
Benefits of research	85	80	χ = 0.29, df = 1, *P* = 0.78
**Types of research**	**15**	**55**	**χ = 13, df = 1,** ***P*** **<0.001**
Personalised example of research	50	49	χ = 0, df = 1, *P* = 1
**Researchers have been ‘approved’ (by regulatory bodies)**	**8**	**52**	**χ = 16.5, df = 1,** ***P*** **<0.001**
Researchers’ confidentiality	46	36	χ = 9, df = 1, *P* = 0.37
Researchers will identify you from the EHR	65	62	χ = 0.1, df = 1. *P* = 0.82
Researchers may contact you in future	85	94	χ = 2.5, df = 1, *P* = 0.21
**C4C is voluntary**	**46**	**85**	**χ = 17.6, df = 1,** ***P*** **<0.001**
Future studies are voluntary	58	61	χ = 0.09, df = 1, *P* = 0.82
**Service user/carer can un-join the register (change their mind)**	**38**	**70**	**χ = 8.9, df = 1,** ***P*** **<0.001**
Decision will not affect care	18	33	χ = 3.1, df = 1, *P* = 0.09
**Can agree contact arrangements; that is, what/when/how contacted**	**31**	**77**	**χ = 23.7, df = 1,** ***P*** **<0.001**
Ask whether they wish to join the register	85	85	χ = 0, df = 1, *P* = 0.1
Questions and concerns	54	65	χ = 1.1, df = 1, *P* = 0.36
**Who to contact for further information**	**19**	**59**	**χ = 13.1, df = 1,** ***P*** **<0.001**

C4C, Consent for Contact; df, degrees of freedom; EHR, Electronic Health Record. Bold data are significant.

#### Why or why not join Consent for Contact; service user and carer feedback

Whilst providing feedback to the researchers, service users and carers both implied altruistic motives. This was the most common reason for agreeing to join C4C and was mentioned by service users/carers in 10 interviews. Service users also described research as an opportunity for social contact.

“Well if I can help somebody else.” (Service user #11)

“I felt very privileged to be asked. It’s something … what was at the most in my mind was the feeling of being useful and contributing something to society.” (Service user #8)

“Yes because I agree in research you can do anything to bring new insight into medicines or into the cure or into the anything. […] We’re happy to participate.” (Carer #17)

“I just automatically said yes because it’d be someone to talk to.” (Service user #4)

“I like research you know it’s someone to talk to, I don’t mind that.” (Service user #16)

Few service users in this study refused to join C4C, but amongst those who did the reasons for refusing included the avoidance of unwanted confusion or hassle. One service user wanted more time to consider before making a decision. One service user stated that they did not want people to access their record or were concerned about confidentiality:

“I don’t want any aggravation.” (Service user #27)

“No I don’t do because I can’t concentrate, you know?” (Service user #23)

“You, you don’t know whether you’re coming or going or whether these people are. […] Genuine. They pass the book onto somebody else to read.” (Service user #10)

## Discussion

This study describes the implementation of C4C within older adults’ mental health services. Focus groups indicated the importance of reassuring service users and carers that they can opt out of future studies, and of considering carers’ commitments. In reality, clinicians’ explanations seldom mentioned the freedom to leave C4C, or that service users/carers could decide how and when they would like to be contacted. The latter was related to whether they agreed to join. Given the increased prevalence of nuisance telephone calls made to older adults [9], it makes sense that service users and carers are encouraged to join based on knowing that they control this aspect of the research contacts.

Clinicians from older adults’ mental health services gave simpler explanations than clinicians from younger adults’ mental health services. They omitted information on the types of research available, or on how the C4C register was governed. Such omissions are understandable; neither concept was mentioned at length by service users or carers within focus groups, and a concise explanation may be preferable. However, leaving out one piece of information – whether service users/carers could stipulate how, when, and how often to be contacted – was related to agreement to join the register and thus needs emphasis. In this study we did not evaluate the effect that providing an information leaflet to service users/carers might have on understanding at a later date.

Clinicians described concerns about explaining C4C where the service user lacked capacity. These concerns are reasonable; clinicians are charged with identifying third-party decision-makers in accordance with the Mental Capacity Act [10]. The NHS constitution states that the public should be informed about relevant research opportunities [1], but clinicians may not view these as being in service users’ best interests. Service users in this study cited altruism and social benefits as the most common reasons for joining C4C. Decision-making amongst people with moderate symptoms of dementia often depends on personal values and relationships [11], so learning about research opportunities may be in service users’ best interests.

## Conclusions

Implementing C4C in older adults’ mental health services requires clinicians to deliver concise, simple explanations that emphasise freedom over how, when, and how often service users and carers can be contacted as well as an explanation of the framework of C4C. The input of carers in decision-making processes may alleviate service users’ anxieties, but carers’ time commitments should be appreciated. These are ways in which clinicians’ explanations of C4C can be adapted to facilitate recruitment in this population.

## Abbreviations

C4C: Consent for Contact
NHS: National Health Service
